# Islet1-expressing cardiac progenitor cells: a comparison across species

**DOI:** 10.1007/s00427-012-0400-1

**Published:** 2012-04-24

**Authors:** Petra Pandur, Ioan Ovidiu Sirbu, Susanne J. Kühl, Melanie Philipp, Michael Kühl

**Affiliations:** Institute for Biochemistry and Molecular Biology, Ulm University, Albert-Einstein-Allee 11, 89081 Ulm, Germany

**Keywords:** Islet1, Heart development, Cardiac progenitor cell, Cardiac stem cell

## Abstract

Adult mammalian cardiac stem cells express the LIM-homeodomain transcription factor Islet1 (Isl1). They are considered remnants of Isl1-positive embryonic cardiac progenitor cells. During amniote heart development, Isl1-positive progenitor cells give rise mainly to the outflow tract, the right ventricle, and parts of the atria. This led to the hypothesis that the development of the right ventricle of the amniote heart depends on the recruitment of additional cells to the primary heart tube. The region from which these additional, Isl1-positive cells originate is called second heart field, as opposed to the first heart field whose cells form the primary heart tube. Here, we review the available data about Isl1 in different species, demonstrating that Isl1 is an important component of the core transcription factor network driving early cardiogenesis in animals of the two clades, deuterostomes, and protostomes. The data support the view of a single cardiac progenitor cell population that includes Isl1-expressing cells and which differentiates into the various cardiac lineages during embryonic development in vertebrates but not in other phyla of the animal kingdom.

**Electronic supplementary material:**

The online version of this article (doi:10.1007/s00427-012-0400-1) contains supplementary material, which is available to authorized users.

## Evolution of the heart

As their size increased, animals have developed pumping organs to ensure proper distribution of oxygen and nutrients throughout the body. In principle, there are two types of circulatory systems: Blood can either flow in a closed vascular network (e.g., in annelids and chordates), or it can be released into the body cavity, such as the hemolymph in arthropods. The morphology of the different circulatory pumps ranges from simple, pulsating (peristaltic) vessels, and tubular hearts, to the more complex multi-chambered hearts (Xavier-Neto et al. 2010). The existence of a contractile organ in both bilaterian clades together with molecular phylogenetic analyses suggests that the common ancestor of protostomes and deuterostomes may have had a simple pumping organ. In this review, we summarize the characteristics of pumping organs of selected developmental and evolutionary biology model organisms focusing on those organisms in which an Isl1-positive cell population has been identified. For more in depth data regarding the evolution of the four-chambered heart, we refer the reader to a recent review by Xavier-Neto et al. (2010).

The closed circulatory system has evolved in coelomates in which the body cavity is lined by a layer of epithelial cells, the mesothelium. In annelids, for example, parts of the mesothelium form the contractile vessels, and no specialized cell types, such as endothelial cells and cardiomyocytes have been identified (Jamieson 1992; Hartenstein and Mandal 2006). In fact, the mesothelium lines the blood vessels in most invertebrate coelomates and, through its ability to contract, causes blood circulation (Hartenstein and Mandal 2006). The dorsal vessel of *Drosophila melanogaster* consists of contractile myoepithelial cells and is similar to the contractile blood vessels of annelids. It was suggested to consider the dorsal vessel of the fly as a “differentiated mesothelium” (Hartenstein and Mandal 2006). This is interesting because there is a strong relationship between the invertebrate mesothelium and the vertebrate vascular system, the latter being derived from the splanchnopleura, the inner layer of the vertebrate mesothelium. This renders the *Drosophila* heart more similar to a vertebrate blood vessel. The heart of molluscs, which also belong to the protostomes, exceeds the simple morphology seen in most protostomes. The mollusc heart generally has two chambers; however, cephalopods have the most complex heart among molluscs consisting of two atria and one ventricle (Budelmann et al. 1997). Studying the molecular mechanisms of cardiogenesis in molluscs would provide valuable insights regarding the degree of conservation of the molecular network between protostomes and deuterostomes that leads to a multi-chambered heart. However, whether the subdivisions of the mollusc heart are homologous to those of vertebrates remains an interesting open issue.

In contrast to the contractile organs present in protostomes, the morphology of the heart in deuterostomes became more complex the higher the demands on oxygen distribution became. Tunicates have a V-shaped contracting vessel, and thus, the pumping organ roughly resembles the heart morphology of the protostomes. The lamprey (agnatha) heart consists of four consecutive compartments: the sinus venosus, the atrium, the ventricle, and the conus arteriosus (Kokubo et al. 2010). In teleosts, the two cardiac chambers, ventricle and atrium, are distinguished by the expression of chamber-specific genes, such as specific myosin heavy chains (Scott and Yelon 2010). The transition from aquatic life to terrestrial life of tetrapods required the development of a more sophisticated and more effective circulatory system, including the appearance of a pulmonary circulatory system. Amphibians serve as a good example for animals whose life cycle is partly aquatic and terrestrial. The heart of *Xenopus laevis* consists of two atria and a single, partially septated ventricle which pumps blood into both the pulmonary and the systemic circuit. Amniotes are characterized by the transition to the fully separated heart with four chambers as found in crocodiles, avians, and mammals (Koshiba-Takeuchi et al. 2009). These more complex, chambered hearts are composed of differently differentiated cell types. Contractile cardiomyocytes have replaced contractile mesothelial cells, and endocardial cells line the inner surface of the heart. The blood vessels are composed of endothelial and smooth muscle cells. Thus, in vertebrates, a variety of specialized cell types has replaced the “cardiac” mesothelium that forms the pumping organs in annelids and arthropods.

**Fig. 1:**
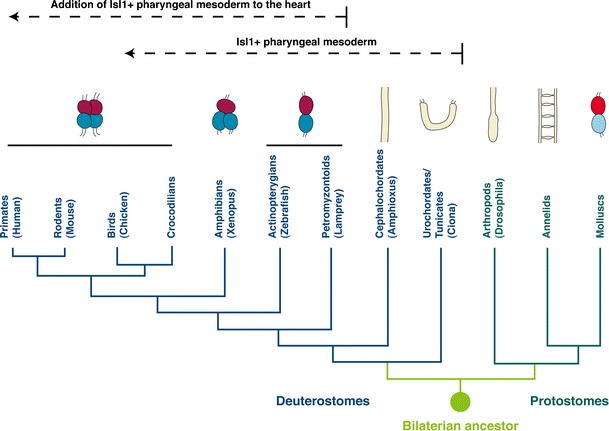
The evolutionary relationship of the organisms discussed in this review and a schematic presentation of the gross morphology of their pumping organs. Ventricular chambers are depicted in *red*; atrial chambers are shown in *blue*. The atrium and ventricle in molluscs are colored in *light blue* and *light red* since it is not clear whether they are indeed homologous to their vertebrate counterparts. The *dashed arrows* indicate the appearance of Isl1-positive pharyngeal mesodermal cells in deuterostomes that contribute to the head musculature and, in craniata, to the heart. The phylogenetic tree is based on Simoes-Costa et al. (2005)

Figure 1 provides a simplified overview of the evolutionary relationships of the animals discussed in this review and the composition of their hearts. These contracting organs of different architecture may have evolved by parallelism or may represent homologous entities that share a common evolutionary origin. The finding of a core set of ortholog transcription factors active during the embryonic development of the hearts in different species suggests that these contracting organs of different architecture share a common evolutionary origin rather than having evolved by parallelism.

## A core set of cardiac transcription factors

A wealth of data from different model organisms has allowed us to identify a conserved core set of transcription factors that is essential for the specification of cardiomyocytes. The bHLH transcription factor Mesp1 is considered to be the earliest marker gene in the specification process of cardiac progenitor cells in vertebrate embryos. The closest Mesp1 ortholog in *Drosophila*, *sage*, seems to be exclusively expressed in the salivary glands and is therefore unlikely to play a role in fly cardiogenesis (Moore et al. 2000). However, it is tempting to speculate whether *sage* holds a function in salivary gland migration. If so, it would indicate a partial functional overlap of Sage and Mesp1, since Mesp1 is required for proper cardiac mesoderm migration in mouse embryos (Saga et al. 1999). However, the important role of Mesp1 in cardiovascular progenitor specification is a characteristic of heart development in deuterostomes.

Additional transcription factors required for cardiogenesis in protostomes and deuterostomes include members of the Nk family of homeobox transcription factors with Nkx2.5 being the factor with the most prominent role in cardiogenesis. Orthologs of Nkx2.5 with a crucial role in cardiogenesis are present in the annelid *Platynereis* (PduNK4), in *Ciona* (Ci-Nkx) and in *Drosophila* (Tinman) (Saudemont et al. 2008; Wada et al. 2003; Bodmer 1993). Regarding the GATA family of transcription factors, there are three members in vertebrates, GATA4, 5, and 6 that have distinct as well as partially redundant functions in cardiogenesis whereas there is only one GATA factor present in *Ciona* (Ci-GATAa) and in *Drosophila* (Pannier) that functions in cardiac development (Sirbu and Pandur 2009; Gajewski et al. 2001; Davidson 2007). Additionally, members of the T-Box family such as Tbx1, 2, 3, 4, 5, 18, and 20 in vertebrates, and Dorsocross1–3 (Doc) (which are related to the vertebrate Tbx6 subfamily) in *Drosophila* are required to ensure proper cardiogenesis (Stennard and Harvey 2005; Reim et al. 2003).

**Fig. 2:**
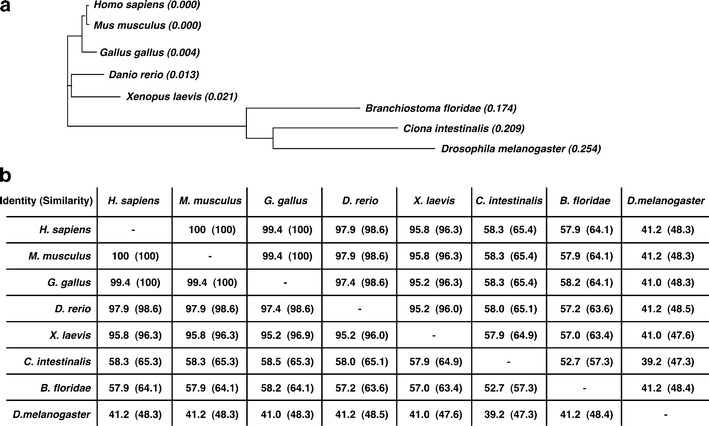
Illustration of the evolutionary relationship of Isl1 genes in different species. **a** The phylogenetic tree was generated using Clustal W (http://www.ebi.ac.uk/Tools/phylogeny/clustalw2_phylogeny/) (Chenna et al. 2003). Numbers indicate the genetic distance between the nodes of the tree. Sequences used were: NP_002193.2 (*Homo sapiens*)**,** NP_067434.3 (*M*. *musculus*), NP_571037.1 (*D*. *rerio*), NP_990745.1 (*G*. *gallus*), NP_001104188.1 (*X*. *laevis*), AF226616_1 (*Branchiostoma floridae*), NP_001027767.1 (*C*. *intestinalis*), NP_476775.1 (*D*. *melanogaster*). **b** Comparison of Isl1 protein sequences. The percentages depict the degree of identical and similar (in *parentheses*) amino acids

Orthologs of the LIM-homeodomain transcription factor Isl1 have been described in humans (Bu et al. 2009), mice (Cai et al. 2003), chicken (Yuan and Schoenwolf 2000), *Xenopus* (Brade et al. 2007; Gessert and Kühl 2009), zebrafish (Hami et al. 2011), lamprey (Kokubo et al. 2010), *Amphioxus* (Jackman et al. 2000), *Ciona* (Stolfi et al. 2010), and *Drosophila* (Mann et al. 2009; Tao et al. 2007). The evolutionary relationships of Isl1 in the different organisms are depicted in Fig. 2. The phylogenetic tree (Fig. 2a) and the accompanying comparison of amino acid sequences of previously characterized Isl1 proteins (Fig. 2b) reveal that invertebrate Isl1 is highly divergent from vertebrate Isl1. While this finding may not come as a surprise, it is interesting that Isl1 proteins among invertebrates are equally highly divergent. However, an alignment of the amino acid sequences reveals the remarkable high conservation of amino acids in the functional domains, that is, the two LIM domains and the homeodomain (Electronic supplementary material Fig. 1). Isl1 was demonstrated to play a role in heart development in all organisms listed above with the exception of *Amphioxus* and annelids where a possible cardiogenic role of Isl1 has not been studied so far. The presence of Isl1-positive adult stem cells in mice and humans has initiated research into a possible function of Isl1 in cardiac repair and regeneration (Moretti et al. 2007). It was demonstrated that these Isl1-positive cardiac stem cells have the capacity to differentiate into functional cardiomyocytes in vitro. Moreover, during cardiac regeneration in urodeles, Isl1 expression is reactivated emphasizing the importance of this transcription factor for cardiomyocyte formation (Witman et al. 2011). Here, we provide an overview of the expression and function of Isl1 during cardiogenesis in different species.

## Expression and function of Isl1 during heart development across species

### *Mus musculus*

Cardiovascular cells originate from *Brachyury*-positive cells in the mesoderm of mouse embryos which start expressing *Mesp1* at early precardiac/primitive streak stages (Solloway and Harvey 2003). *Mesp1*-positive cells are heterogeneous in fate, as they are able to give rise to all cardiac cells, skeletal muscles of the head and neck, and paraxial mesoderm derivatives (Saga et al. 1999). As soon as these cells leave the primitive streak and populate the anterior and lateral plate mesoderm to form the cardiac crescent, they turn off *Mesp1* and become irreversibly committed to a cardiac fate by expressing *Nkx2.5*. Further differential expression of *Tbx5* and *Isl1* subdivides the cardiac crescent into two distinct domains, the first heart field (FHF) and the second heart field (SHF). As the cells of the FHF give rise to the left ventricle and parts of the right ventricle and atrium and as the cells of the SHF give rise to the right ventricle, outflow tract, and parts of both atria, the term lineage is more expedient and also used in this context. Expression of *Tbx5* and *Isl1* also marks an important restriction in the differentiation potency of cardiac progenitors, since these cells differentiate mainly to cardiomyocyte and smooth muscle vascular cells and, to a lesser extent, to endothelial cells (Watanabe and Buckingham 2010). Irreversible genetic marking of progenitor cells using the *Isl1–Cre;Rosa26* ^*fsLz*^ system showed that *Isl1* ^*+*^ progenitors contribute to cardiomyocyte, smooth muscle, and endothelial cells in the right ventricle, outflow tract, portions of the atria, and the inner curvature of the left ventricle (Cai et al. 2003; Sun et al. 2007). These studies are in accordance with the phenotype of *Isl1* ^-/-^ mouse embryos, which die at midgestation due to a severely altered heart characterized by a single ventricular chamber with left ventricle identity, absence of outflow tract and of the right ventricle, and a severe reduction of atria (Cai et al. 2003).

**Fig. 3:**
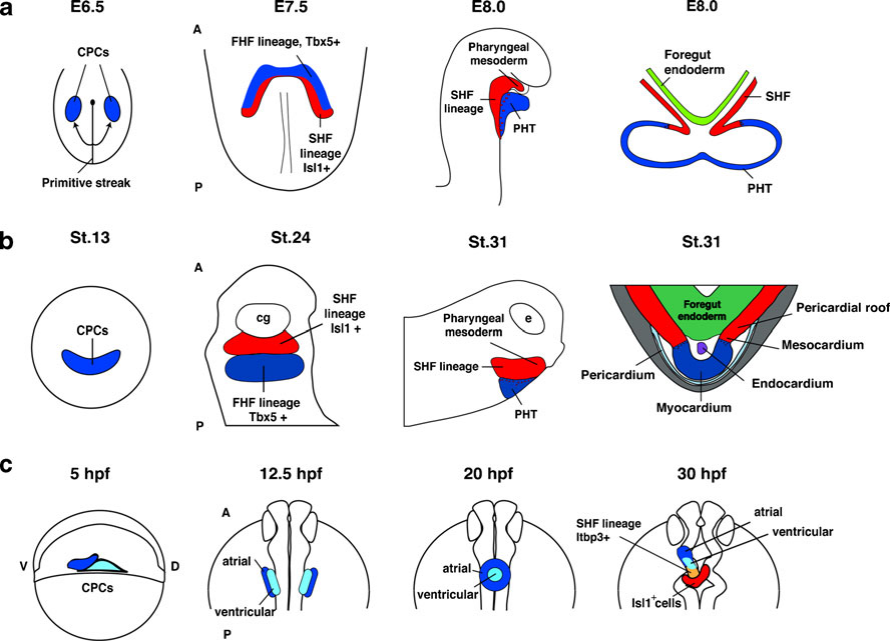
A simplified schematic presentation of heart development in different species. **a** Mouse. Developmental stages are indicated in embryonic days (E). The cartoons show a dorsal view of a flattened embryo (E6.5), an anterior view (E7.5), and a lateral view (E8.0). Additionally, a schematic illustration of a transverse section at E8.0 is provided. The SHF is characterized by the presence of Isl1-positive cells and is indicated in *red*. **b** *X*. *laevis*. Developmental stages are according to Nieuwkoop and Faber (1975). The *cartoons* show an anterior view (st.13), a ventral view (st.24), and a lateral view (st.31). Additionally, a schematic illustration of a transverse section through the heart at st.31 is provided. At st.24, cells of the SHF (*red*) express *isl1* whereas cells of the FHF (*blue*) are positive for *tbx5*. **c** *D*. *rerio*. Developmental stages are indicated in hours post-fertilization (hpf). The cartoons show a lateral view for stage 5 hpf and dorsal views for stages 12.5, 20, and 30 hpf with the anterior side up. The localization of early cardiac progenitor cells at 40 % epiboly, as well as the distinction between an atrial and a ventricular fate was analyzed by fate-mapping experiments. An Isl1-positive cell population that contributes to the arterial pole is located posterior and dorsal to the ventricle and is indicated in *red*. It only partially contributes to the heart. The zebrafish SHF is characterized by the expression of *ltbp3*. Of note, the arrangement of the atrium and ventricle along the anterior–posterior axis is inverted at later stages (not shown here). As a result, the atrium will come to lie posterior to the ventricle. *CPC*: common cardiac progenitor cell population, *A*: anterior, *D*: dorsal, *P*: posterior, *V*: ventral, *FHF*: first heart field; *SHF*: second heart field, *PHT*: primary heart tube. Partly adopted from Gessert and Kühl (2009)

The SHF is situated medial, slightly caudal, and dorsal to the first heart field, in the so-called pharyngeal mesoderm (Fig. 3a). During heart looping stages, *Isl1*-positive cells from the pharyngeal mesoderm are continuously added to the poles of the embryonic heart (Waldo et al. 2001; Mjaatvedt et al. 2001). Of note, the pharyngeal cardiogenic mesoderm has not only cardiogenic but also skeletal myogenic potential and strongly contributes to the development of the head muscles derived from the first and second branchial arches (with a lesser extent to the masseter, pterygoid, and temporalis). This finding has led not only to the description of the so-called cardio-craniofacial field of multipotent cardio-skeletal progenitors but also to a better understanding of the intricate relationship between pharyngeal muscles and cardiac development during evolution (Tirosh-Finkel et al. 2006; Lescroart et al. 2010; Tzahor and Evans 2011; Nathan et al. 2008).

Transcription of *Isl1* is shut off as soon as the progenitor cells enter the forming embryonic heart per se, suggesting that Isl1 is required for continuous proliferation, expansion, and migration of cardiovascular precursors. However, *Isl1* ^+^ cells persist in the hearts of fetal and young (up to 10 months old) adult rodents, in quiescent cells with either cardiomyocyte or peripheral nervous ganglia characteristics (Genead et al. 2010; Laugwitz et al. 2005; Khattar et al. 2011).

Interestingly, a population of Isl1-positive adult cardiac stem cells can be identified in the postnatal heart of mice, rats, and humans (Laugwitz et al. 2005, 2008). These cells can develop into functional cardiomyocytes in vitro as demonstrated by marker gene expression, intracellular calcium release, and their ability to initiate action potentials (Laugwitz et al. 2005). A possible involvement of these cells in the recently described replacement of adult cardiomyocytes in vertebrates awaits further investigation (for review, see Kühl and Kühl 2011). In general, the Isl1-positive adult cardiac stem cells are detected in regions of the postnatal heart that originated from the SHF and thus are considered to be remnants of the aforementioned Isl1-positive cells present during the development of the heart.

### *Gallus gallus*

In chicken, the cardiac progenitors migrate from the cranial primitive streak (Hamburger Hamilton stage 3, HH3) rostrally to the lateral plate mesoderm (HH6) where they irreversibly differentiate towards a cardiac identity by expressing Nkx2.5 and GATA4 (Garcia-Martinez and Schoenwolf 1993; Schultheiss et al. 1995). The two lateral plate cardiogenic areas come together and fuse at the midline at stage HH8–9 to form the heart tube around HH10 (Abu-Issa and Kirby (2007) and references therein). The newly formed heart tube is basically non-proliferating, and its growth requires continuous addition of cells to the anterior and posterior poles from the second heart field situated dorsally in the pharyngeal mesoderm (Waldo et al. 2001; Mjaatvedt et al. 2001; Tzahor and Evans 2011). Note that the first description of the second heart field in chicken used the term anterior heart field and only described a subpopulation of the SHF contributing to the arterial pole of the heart. The contribution of cells from the second heart field to the heart primordium is exhausted around HH18 (Waldo et al. 2001) and correlates with cells expressing *Isl1* (van den Berg and Moorman 2009). Recent experiments have demonstrated that the chicken pharyngeal mesoderm also contributes to the pharyngeal muscles (Tirosh-Finkel et al. 2006; Nathan et al. 2008). Similar to *Xenopus* embryos (see below), the expression of chicken *Isl1* is peculiar since it initially (HH4) overlaps with the entire cardiogenic area (Yuan and Schoenwolf 2000; Brade et al. 2007). The function of Isl1 during chicken heart development has not been studied so far. For example, it would be interesting to see whether Isl1 also plays a crucial role in the formation of the right ventricle of the four-chambered chicken heart as it does in the mouse.

### *X. laevis*

*X*. *laevis* has a three-chambered heart consisting of one partially septated ventricle and two atria. The expression and function of *isl1* during *Xenopus* cardiac development has been analyzed in two previous studies (Brade et al. 2007; Gessert and Kühl 2009). *Isl1* expression is first detected at the end of gastrulation (Nieuwkoop Faber stage 13) in the anterior ventral mesoderm of *Xenopus* embryos by whole-mount in situ hybridization (Gessert and Kühl 2009; Brade et al. 2007). At this stage, *isl1* is coexpressed with other cardiac transcription factors such as *nkx2.5, gata6,* or *tbx1,* although expression data on a single cell level are not yet available. To analyze the fate of these *isl1*-positive cells in *Xenopus*, fate-mapping experiments were performed using DiI as a lineage tracer. To this end, a small group of cells in the anterior ventral region of stage 13 *Xenopus* embryos was labeled by the injection of DiI, and the fate of these cells was followed during further development (Gessert and Kühl 2009). Cells of this region contribute to all parts of the tadpole *Xenopus* heart when the different components of the heart, the outflow tract, the ventricle, the atrium, and the inflow tract can be distinguished. Of note, the atrium is not yet septated at this stage. Both sets of data, the expression pattern and fate mapping results, strongly indicate that, in *Xenopus*, *isl1*-expressing cells are part of a common cardiac progenitor cell (CPC) population.

In situ hybridization studies further demonstrated that, starting at early tailbud stages, the CPC population separates into an *isl1*-positive anterior domain and a *tbx5*-positive posterior domain (Fig. 3b; Gessert and Kühl 2009). These data could also be supported by single-cell RT-PCR experiments performed at stage 24 in which cardiac cells could be identified being either positive for *isl1* and *nkx2.5* or *tbx5* and *nkx2.5* (Gessert and Kühl 2009). Additionally, *isl1* expression could be detected in the pharyngeal mesoderm and in the mesenchyme of the pharyngeal pouches, similar to *isl1* expression in the mouse (Brade et al. 2007). Consistent with this expression pattern, labeling of the anterior *isl1*-positive cells at stage 24 showed that these cells contribute to the aortic arch arteries, the aortic sac, the distal and proximal outflow tract (OFT), and also to the jaw muscles (Lee and Saint-Jeannet 2011; Gessert and Kühl 2009). This suggests the existence of cells with SHF lineage properties in *Xenopus* comparable to the SHF lineage described in chicken and mouse (Fig. 3b). Labeling of cells located more posterior to the *isl1*-positive cell population, which express *nkx2.5* and *tbx5* resulted in DiI-positive cells in the ventricle, the atrium, and the inflow tract, which may be called a FHF lineage. Thus, the separation into FHF and SHF lineages in *Xenopus* is indicated by the differential expression of *isl1* and *tbx5* which resembles the situation in the mouse.

When the heart tube starts to form, *isl1* expression within the heart tube is restricted to the dorsolateral regions, the mesocardium, and pericardial roof. *Isl1* transcripts are absent from the differentiating myocardium that starts to express the cardiomyocyte differentiation marker *troponin Ic* (Gessert and Kühl 2009; Brade et al. 2007). This *isl1* expression pattern led to the hypothesis that the *isl1*-positive cells are added to the growing heart tube when it starts to undergo looping to establish the different compartments of the heart as shown in mouse and chicken. However, detailed experimental evidence for this hypothesis is still missing.

To interfere with isl1 function during *Xenopus* cardiac development, an *isl1 antisense* morpholino oligonucleotide (MO) was unilaterally injected into the dorsal marginal zone region of early cleavage-stage embryos (Brade et al. 2007). MO-induced knock-down of isl1 results in severe heart abnormalities during development. Dissected hearts revealed a significant size reduction of isl1 MO-injected hearts compared with untreated hearts. In some embryos, the heart tube failed to undergo looping. Molecular analyses showed that expression of core members of the cardiac transcriptional network, e.g., *gata 4, gata 6, nkx2.5*, and *tbx20*, are downregulated after isl1 MO injection. Taken together, these findings clearly indicate that *isl1* is required early in the CPC population to guarantee normal heart development. A potential later role of *isl1* as part of the SHF lineage in *Xenopus* has not been investigated so far and would require the design of hormone-inducible *isl1* constructs which, in contrast to MO-mediated knock-down experiments, allow for a temporal controlled interference.

### *Danio rerio*

The zebrafish heart has two chambers, a ventricle and an atrium. The earliest progenitors of the zebrafish heart are detected bilaterally of the body axis at the onset of gastrulation (5 h post-fertilization (hpf); Fig. 3c) (Lee et al. 1994; Scott and Yelon 2010). These cardiac progenitor cell populations will fuse to form the cardiac cone around 21 somites stage (21ss). This cone undergoes a process called cardiac jogging which finally results in a bent, two-chambered primary heart tube at 48 hpf. Cardiac differentiation is already initiated at segmentation stages when the early cardiac progenitors are still located bilaterally to the midline of the embryo (Schoenebeck and Yelon 2007). The first sign of differentiation is the onset of *cardiac myosin light chain 2* (*cmlc2*) expression at 14ss. Myocardial cell differentiation commences in cells that will later form the ventricle. Differentiation continues in cells of the atrium and subsequently extends to cells of the venous pole. Cells at the arterial pole will differentiate at a different time point (de Pater et al. 2009).

In zebrafish, expression of Isl1 can be detected by immunohistochemistry from early somite stages on, however, the predominant expression domains are neural structures (Korzh et al. 1993). The neural expression was confirmed by using a transgenic zebrafish line expressing GFP under the control of the *isl1* promoter (Higashijima et al. 2000). The analysis of *isl1* expression in cardiac lineages, however, is difficult in this transgenic reporter line. Thus, it has been assumed for a long time that *isl1* is not expressed in cardiac precursor cells in zebrafish.

Two recent studies also employed immunohistochemistry techniques and do provide evidence for Isl1 expression in the heart of zebrafish (de Pater et al. 2009; Hami et al. 2011). From 14ss on, low expression of Isl1 is detected in an area ventral and lateral to the trigeminal placode, similar to the expression domain of GFP in *cmlc2*::GFP transgenic fish in which GFP is expressed under the control of the *cardiac myosin light chain 2* promoter (Hami et al. 2011). Analyses of *isl1* expression on sections show that *isl1* is also present in the splanchnic mesoderm adjacent to the cardiac mesoderm and in a few cardiomyocyte progenitors located in a region which gives rise to the arterial pole at later stages. At 23ss, Isl1 is localized in vascular endothelium, in the endoderm dorsal to the heart cone and in a subset of cardiomyocytes (de Pater et al. 2009). This demonstrates that Isl1 is expressed in cardiac cells, when the heart cone is forming. Additionally, Isl1 is detected in the mesoderm, where yet undifferentiated heart cells are residing. Starting around 24 hpf (approximately 30ss), Isl1-positive cells are present in the mesenchyme of the branchial arches next to the future arterial pole. This expression persists during heart tube extension and looping and ceases soon thereafter (de Pater et al. 2009). In summary, Isl1 is expressed in a population of cells that can be considered to be equivalent to the SHF in other species. This is also confirmed by lineage labeling experiments showing a contribution of Isl1-positive cells to the arterial pole of the heart (Hami et al. 2011). It remains unclear, however, whether Isl1 is also expressed earlier in a common CPC population in zebrafish.

Given the new insight that Isl1 is also expressed in cardiac progenitor cells, the potential function of Isl1 in zebrafish cardiogenesis was recently studied. Using ENU mutagenesis, an *isl1* mutant fish line was generated (*islK88X*) (de Pater et al. 2009). In this mutant, a single base mutation results in a premature stop codon, which generates a truncated Isl1 protein that lacks the entire homeodomain and part of the LIM domains. Regarding the overall appearance, *isl1* mutant fish are initially indistinguishable from wild-type siblings, except for being immotile, most probably due to a dysfunction of motor neurons. Isl1 mutant fish die between 5 and 7 days post-fertilization. Closer analysis of the heart revealed that loss of Isl1 function results in arrhythmia as well as bradycardia. However, in contrast to the findings in *Xenopus*, neither a knock-down of Isl1 in zebrafish using morpholino oligonucleotides nor the *isl1* mutant line exhibited a downregulation of early cardiac markers, e.g., *nkx2.5* (de Pater et al. 2009). Although *isl1* mutant embryos developed substantially fewer cardiomyocytes at the venous pole, no obvious phenotype was observed at the arterial pole as could have been expected from the Isl1 expression. Most importantly, the authors already point out that this finding may indicate an evolutionary difference between teleosts and amniotes with respect to recruiting cells to the venous and the arterial pole, respectively. Unlike in the mouse where Isl1 is required for the recruitment of cells to both poles of the forming heart (Cai et al. 2003), the function of zebrafish Isl1 appears to be more restricted.

This may be explained by the fact that cardiomyocyte differentiation at the arterial pole does not require Isl1 (de Pater et al. 2009) which leaves Isl1 with the role of a molecular marker for the SHF in fish rather than an essential functional component. This notion is substantiated by a recent publication ascribing a functional role for *latent TGF-β binding protein 3* (*ltbp3*) in zebrafish SHF development (Zhou et al. 2011). The authors describe similarities between the *ltbp3* morphant phenotype and the SHF phenotypes in *isl1* mutant mouse embryos. This important piece of data indicates that the crucial role of mammalian *isl1* in the SHF lineage is taken on by *ltbp3* in zebrafish. Nevertheless, the phenotype of *isl1* mutant fish is interesting as parts of the heart are affected for which no *isl1* expression has been demonstrated so far.

### *Ciona intestinalis*

Tunicates are chordates and represent a group of sessile marine invertebrates. Based on extensive molecular phylogenetic studies, they are considered to represent the sister group of vertebrates in the phylum of chordates (Delsuc et al. 2006; Bourlat et al. 2006; Blair et al. 2005). *Ciona* larvae develop characteristic features of chordates including a notochord, a nerve chord, and a tail. During metamorphosis, the tail degenerates and the adult organs are formed from the remaining larval structures of the head. This also includes the heart, which consists of a simple V-shaped myocardial tube devoid of an endocardial layer, located in a pericardial sac. At either end, the myocardial tube connects to two vessels located on the dorsal and the ventral body side, respectively, feeding in an open circulatory system. The myocardial tube and the vessels are connected by a group of undifferentiated, proliferating, non-contracting cells, termed growth zone. The myocardial tube is lined with a sheet of undifferentiated cells, which form the so-called undifferentiated line. The *Ciona* heart shows the exciting property of life-long regeneration with new cardiomyocytes forming from the cells of the growth zone and possibly also from the undifferentiated line. For a more detailed description of *Ciona* heart development, we refer the reader to recent reviews (Davidson 2007; Levine 2010).

**Fig. 4:**
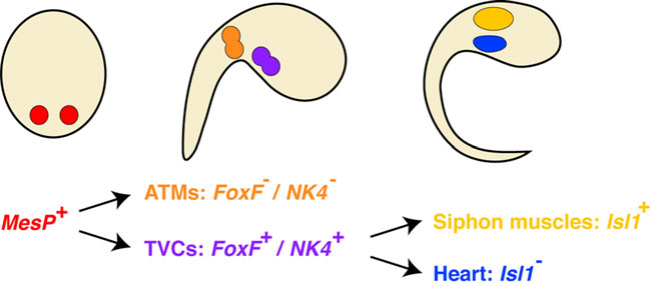
Early heart development in *C*. *intestinalis.* The heart derives from the bilateral B7.5 blastomeres indicated in *red*. *TVC*: trunk ventral cells, *ATM*: anterior tail muscles. Adopted from Stolfi et al. (2010)

Cell fate-mapping in *Ciona* demonstrated that the cardiac lineage derives from the bilaterally located B7.5 cells (Fig. 4), which express *Ci-Mesp*, the *Ciona* Mesp1 ortholog (Satou et al. 2004). The more anteriorly located progeny of the B7.5 cells represent the trunk ventral cells (TVCs) that give rise to the heart, whereas the more posteriorly located progeny of the B7.5 cells will form the anterior tail muscles (Fig. 4). Consistent with their cardiac fate, the trunk ventral cells express the transcription factors NK4 (the Tinman/Nkx2.5 ortholog), FoxF, GATAa, and Hand-like. Descendants of B7.5 cells not only generate the heart as indicated by a MesP > H2B::CFP transgene but also give rise to the atrial siphon muscle (ASM) after metamorphosis (Stolfi et al. 2010). The two muscle types can be discriminated by the expression of the myosin heavy chain isoforms MHC2 and MHC3 (Ogasawara et al. 2002). A reporter construct driven by a 3.2-kb regulatory region of the single *Ciona isl1* gene is expressed in the ASM in juveniles. Precursor cells of the heart, however, are located more ventrally and medially to the progenitors of the ASM. Interestingly, this arrangement resembles the relative position of cells of the FHF and SHF lineage in vertebrates, but a contribution of Isl1-positive cells to the tunicate heart could not be demonstrated. This finding suggests that both, vertebrates and tunicates, posses a population of cardiopharyngeal mesodermal cells that arises from Mesp1-positive progenitor cells which give rise to jaw and siphon muscles.

### *D*. *melanogaster*

From an evolutionary perspective, it is very interesting that the *Drosophila* ortholog of *isl1*, *tailup* (*tup*), was demonstrated to hold a crucial role in fly heart development. The fly heart, also called the dorsal vessel, is a simple tube that consists in principle of two main cardiac cell types. The contractile myocardial cells form the lumen of the dorsal vessel and are surrounded by a number of pericardial cells. The heart tube is subdivided into the posterior heart proper and the anterior aorta with a valve separating these two “chambers.” Localized bilaterally at the anterior end of the aorta are cell clusters of the forming lymph glands, which are the hematopoietic organ in *Drosophila* larvae. During embryonic heart development, Tup expression is detected in all myocardial cells of the heart tube, in a subset of pericardial cells, as well as in a subset of lymph gland cells. Tup expression is also detected early in the cardiac mesoderm where it co-localizes with some Tinman (Tin)-expressing and Even-skipped (Eve)-positive pericardial cells (Mann et al. 2009). The Tup/Isl1 expression pattern supports a role for this factor in the development of different cardiac lineages. Analyses of *tup* mutant embryos revealed defects for all examined molecular markers for the different heart and lymph gland cell types (Mann et al. 2009; Tao et al. 2007). It could be demonstrated that the early expression of the crucial cardiac factors Tinman (Tin), Pannier (Pnr), and Dorsocross (Doc) was severely affected in *tup* mutants and *vice versa*; Tup expression was strongly downregulated in embryos mutant either for *tin*, *pnr*, or *Doc* (Mann et al. 2009). These findings were the first genetic evidence for the interdependency of essential cardiac factors including *tup*. Moreover, additional experiments set up to demonstrate genetic interactions showed indeed that *tup* and *tin*, as well as *tup* and *pnr* cooperate during cardiogenesis (Mann et al. 2009).

An additional interesting piece of data came from the analysis of the *Drosophila hand* enhancer by Tao et al. (2007). The authors identified and confirmed Tup binding sites in the *hand* enhancer and demonstrated the importance of the presence of these sites for *hand* expression in vivo. This finding is interesting with respect to mouse heart development where the *hand2* gene functions in SHF progenitors and is required for proper cardiogenesis (Tsuchihashi et al. 2011). However, a regulation of *hand2* through Isl1 has not yet been investigated. Taken together, the described data are clear evidence for the requirement of *tup/isl1* in early steps of *Drosophila* cardiogenesis and add *tup/isl1* to the core set of ancestral cardiac transcription factors.

## Evidences for an Isl1-positive common cardiac progenitor

Isl1 was initially hypothesized to be a specific marker for the SHF and to solely function in the development of SHF lineages. However, accumulating data strongly indicate that Isl1 is already expressed in the common cardiac progenitor cell population and has an important function in heart development. In chicken and *Xenopus*, it was clearly demonstrated that *isl1* transcripts are present in this progenitor pool which splits into FHF and SHF lineages during development (Yuan and Schoenwolf 2000; Brade et al. 2007; Abu-Issa and Kirby 2008). Also, as demonstrated by immunohistochemistry, Isl1 expression in mouse embryos commences before E7.5 (Prall et al. 2007) in regions that give rise to both FHF and SHF lineages, providing more evidence for the existence of an initially single heart field. Moreover, by applying a similar *Isl1–Cre* lineage analysis as was performed by Cai et al. (2003) but using a different floxed locus (*Gata* ^*flap*^), it was shown that the overall contribution of *isl1*-positive cells to different heart compartments is much broader (Ma et al. 2008). This finding reinforces the above listed observations that *isl1* marks a much earlier, common cardiovascular progenitor cell as opposed to solely being a marker of the second heart field. Also, results obtained from murine and human embryonic stem (ES) cell cultures indicate that Isl1 is one of the first cardiac transcription factors that marks a common progenitor, which gives rise not only to the FHF and SHF lineages but also to cells of the vascular system. In particular, Isl1-positive progenitors can also develop into endothelial and smooth muscle cells (Moretti et al. 2006, 2010; Bu et al. 2009). Consistently, a knock-down of Isl1 in *Xenopus* not only results in cardiac but also in endothelial marker gene expression defects (Brade et al. 2007). A recent study suggested that an early function of Isl1 is required for endothelial differentiation in ES cells, whereas slightly later Isl1 is required for cardiomyocyte development (Bondue et al. 2011). Importantly, even in *Drosophila*, the *isl1* ortholog *tup* is expressed early in the cardiac mesoderm and loss of function results in heart defects encompassing the different heart cell types as well as lymph gland cells (Mann et al. 2009; Tao et al. 2007). With respect to these comparable findings across species, it is interesting that neither in zebrafish nor in *Amphioxus* or lamprey has Isl1 expression been unambiguously shown to be present in the CPC pool of cells (Jackman et al. 2000; de Pater et al. 2009; Hami et al. 2011; Kokubo et al. 2010). This could be due to technical limitations to detect *isl1* transcripts or Isl1 protein in the cardiac mesoderm of these organisms.

Since, in *Drosophila* and *Xenopus,* Isl1 is expressed in the cardiac mesoderm and was shown to be important for cardiogenesis, several questions arise. Has Isl1 been integrated independently into the cardiac gene regulatory networks of *Drosophila* (or protostomes in general) and of vertebrates? Here, it is of particular interest that the *Drosophila* dorsal vessel could be considered as a specialized blood vessel with the ability to contract (as described above) and which is equally related to vertebrate blood vessels (most probably to veins) and vertebrate hearts. Moreover, since, in vertebrates, Isl1 is not only found in the common cardiac progenitor but also in endothelial and smooth muscle precursor cells, it is tempting to speculate that Isl1 might label a subpopulation of mesothelial cells. Also of interest is the question whether the function of Isl1 during early cardiogenesis became dispensable in tunicates and fishes (and possibly other species). Maybe the role of Isl1 in early cardiac (and vascular) development in tunicates and fishes has not been fully understood so far. Further analyses of these issues will help to clarify these questions.

## Isl1 within a cardiac gene regulatory network

Hardly anything is known about the regulation of *isl1* expression in the common cardiovascular progenitor cell population in vertebrates. Contradicting data are available with respect to a potential regulation of *isl1* through Mesp1. A recent gain and loss of function study suggested that Isl1 is not regulated by Mesp1, blatantly contradicting previous results showing an upregulation of Isl1 expression upon Mesp1 overexpression (Lindsley et al. 2008; Bondue et al. 2011; 2008). This issue therefore is not yet solved. A dual reporter approach described an enhancer, 3–6-kb downstream of the mouse *isl1* locus, that faithfully reproduces *isl1* expression in the heart of E7.5–E10.5 transgenic mouse embryos and contains conserved potential binding sites for Foxo/Foxd, Tcf1, and GATA transcription factors (Kappen and Salbaum 2009). However, investigation of the interactions of *isl1* with the other cardiac core transcription factors in the CPC requires further detailed investigations. In *Drosophila*, however, it has already been shown, as discussed earlier in this review, that *tup/isl1* expression depends on *tin* (*nkx2.5* ortholog), *Doc* (Tbx factor ortholog), and *pnr* (GATA factor ortholog) and *vice versa*. Loss-of-function studies in *Xenopus* also suggest an involvement of Isl1 in the early cardiac regulatory network (Brade et al. 2007). Thus, it is tempting to speculate that these four classes of transcription factors are wired to form a stable gene regulatory network within the common cardiac progenitor cell population.

More is known about the regulation of *isl1* expression in the second heart field in the mouse. The *Isl1*-positive cardiac progenitors in the pharyngeal mesoderm are subjected to regulations from several signaling pathways, including the Wnt, BMP, and FGF signaling pathways. There is a general consensus that Wnt signaling functions at various stages of heart development to promote proliferation of cardiac progenitors and blocking their differentiation (reviewed in Gessert and Kühl 2010). In this context, it is interesting to note that *Isl1* and *FGF10* (both endogenous markers for the second heart field) are direct targets of Wnt/β-catenin signaling (Cohen et al. 2007; Lin et al. 2007; Qyang et al. 2007), implicating Wnt and FGF signaling in the proliferation of *Isl1*-positive progenitors. Furthermore, *FGF8* (an additional endogenous marker for the second heart field) has been shown to be required for the elongation of the heart and development of the right ventricle and the OFT. FGF8 also controls *Isl1* expression in the pharyngeal mesoderm, thus connecting *Isl1* expression not only to FGF signaling but also to retinoic acid and Tbx1 signaling (Sirbu et al. 2008; Ryckebusch et al. 2008; Ilagan et al. 2006; Park et al. 2006; Xu et al. 2004). Notch signaling inhibits Wnt/β-catenin signaling in the cardiac precursors, while the Notch target gene Hes1 is expressed in a pharyngeal domain overlapping with Isl1 expression and is required for proliferation in the second heart field and outflow tract extension, thus integrating Notch signaling in a cardiac differentiation-promoting position within the cardiac transcription network (Kwon et al. 2009; Rochais et al. 2009). Interestingly, Isl1 was found to directly regulate the expression of *Myocd*, a transcriptional cofactor of SRF known to promote the development of ventricular cardiomyocytes to functions as a molecular switch between vascular smooth muscle and skeletal muscle differentiation (Hoofnagle et al. 2011; Wang et al. 2001; Li et al. 2003; Long et al. 2007).

More data on Isl1 regulation have come from the evaluation of mutant mouse embryos phenocopying the *Isl1* ^*-/-*^ heart phenotype. *Mef2c* ^*-/-*^ and *Foxh1* ^*-/-*^ embryos both lack the outflow tract and the right ventricle, with *Mef2c* being a direct target of both Isl1 and Foxh1 (while *Isl1* transcription does not depend on Foxh1) (Dodou et al. 2004; von Both et al. 2004). BMP4 signaling has been shown to be required for OFT development (Liu et al. 2004) while a conditional deletion of the BMP receptor BMPR1a using *Isl1–Cre* affected both the outflow tract and the right ventricle (Yang et al. 2006). Interestingly, *Isl1* expression was found to be upregulated in *Isl1-Cre/BMPR1a* embryos, a negative effect that was subsequently shown to be mediated by microRNAs from the BMP4-regulated *miR-17-92* s cluster (Wang et al. 2010). Last but not least, *shh* mutant embryos are also characterized by defective OFT and SHF development as Shh signaling from the pharyngeal endoderm is required for correct Isl1 expression and for correct development of SHF derivatives (Goddeeris et al. 2007; Washington Smoak et al. 2005; Lin et al. 2006). This network appears to be conserved across species as in zebrafish; Isl1 expression in the second heart field also depends on Shh function and Tbx1 expression (Hami et al. 2011). In summary, these data describe the image of a complex second heart field regulatory network with Isl1 in its center.

## Towards an evolutionary analysis of cardiac gene regulatory networks

**Fig. 5:**
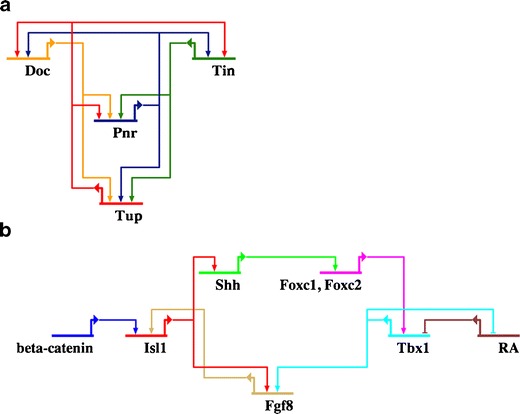
Gene regulatory networks in cardiac cells including Isl1. **a** Interaction of key cardiac transcription factors in the early cardiac mesoderm in *Drosophila*. *doc*: dorsocross, *tup*: tailup, *pnr*: pannier, *tin*: Tinman. **b** Interaction of signaling molecules and transcription factors including Isl1 in the pharyngeal mesoderm/SHF in the mouse. *Shh*: sonic hedgehog, *RA*: retinoic acid, *Fgf8*: fibroblast growth factor 8

Taken together, the data from different organisms suggest two distinct functions of Isl1 during cardiogenesis. Early during heart development, Isl1 is part of the gene regulatory network required for the establishment of a common cardiac progenitor cell population. Data from *Drosophila* and *Xenopus* suggest a conserved network of regulatory interactions involving Isl1 and members of the GATA, Nkx, and Tbx families of transcription factors (Fig. 5a). Likely, a similar network also exists in higher vertebrates, e.g., the mouse. Further studies are required to elucidate these interactions.

In addition, Isl1 plays an important second role in the cardiac development of deuterostomes (Fig. 1). During embryonic development of chordates, Isl1 labels a population of pharyngeal mesodermal cells contributing to the heart as well as to the musculature of the head. In chordates such as *Ciona*, the corresponding cell population only gives rise to the atrial siphon muscle but not to the heart. In craniates such as the zebrafish and *Xenopus*, these cells only contribute to the arterial pole of the heart. In contrast, they also contribute to the venous pole in amniotes. Data from the mouse and the zebrafish, again, argue for a conserved regulatory network in this population of cells involving factors such as Isl1, Tbx1, and Shh (Fig. 5b).

So far, the available data provide significant insight into the gene regulatory networks that govern cardiogenesis. However, more research is needed to obtain a comprehensive understanding of the molecular networks present in the common CPC population and in the pharyngeal mesoderm across species.

## Electronic supplementary material

Below is the link to the electronic supplementary material.

ESM 1 (DOC 46 kb)
