# Supplementary material for: Islet1-expressing cardiac progenitor cells: a comparison across species
Source: Dev Genes Evol. 2012 Apr 24;223(1):117–29. doi: 10.1007/s00427-012-0400-1 (PMC3552366; doi:10.1007/s00427-012-0400-1)
Supplement: Supplementary file 1 — (DOC 46 kb) [file 427_2012_400_MOESM1_ESM.doc]

**Supplementary Material**

**Islet1 expressing cardiac progenitor cells: A comparison across species**

Petra Pandur, Ioan Ovidiu Sirbu, Susanne J. Kühl, Melanie Philipp, Michael Kühl*

Institute for Biochemistry and Molecular Biology, Ulm University, Albert-Einstein-Allee 11, D-89081 Ulm, Germany

*** Author for correspondence:**

Michael Kühl

Institute for Biochemistry and Molecular Biology

Ulm University

Albert-Einstein-Allee 11

D-89081 Ulm, Germany

Phone: ++49-731-500-23283

Fax: ++49-731-500-23277

Email: [michael.kuehl@uni-ulm.de](mailto:michael.kuehl@uni-ulm.de)

**Suppl. Figure 1:** Alignment of Isl1 proteins of different species. The LIM domains are indicated in green and blue, respectively, the homeodomain is given in yellow. Red letters indicate the Zn-binding sites.

Isl1 alignement

Homo_sapiens -----------------------------------MGDMG--DPPKKKRLISLCVGCGNQ

Mus_musculus -----------------------------------MGDMG--DPPKKKRLISLCVGCGNQ

Danio_rerio -----------------------------------MGDMG--DPPKKKRLISLCVGCGNQ

Gallus_gallus -----------------------------------MGDMG--DPPKKKRLISLCVGCGNQ

Xenopus_laevis -----------------------------------MGDMG--DPPKKKRLISLCVGCGNQ

Branchiostoma_floridae -------------------------------------MPG--DPPKKNRRNAMCVGCGSH

Ciona_intestinalis -----------------------------------MEAACRPRLIATPCRIPLCVGCGSP

Drosophila_melanogaster MVMAEIGGHLAHQLPLHNHNHNQTGLQPSLVMNHHLDLDCHGHDVIKKQRLSHCVGCGGQ

Homo_sapiens IHDQYILRVSPDLEWHAACLKCAECNQYLDESCTCFVRDGKTYCKRDYIRLYGIKCAKCS

Mus_musculus IHDQYILRVSPDLEWHAACLKCAECNQYLDESCTCFVRDGKTYCKRDYIRLYGIKCAKCS

Danio_rerio IHDQYILRVSPDLEWHAACLKCAECNQYLDESCTCFVRDGKTYCKRDYIRLYGIKCAKCN

Gallus_gallus IHDQYILRVSPDLEWHAACLKCAECNQYLDETCTCFVRDGKTYCKRDYIRLYGIKCAKCS

Xenopus_laevis IHDQYILRVSPDLEWHAACLKCAECSQYLDESCTCFVRDGKTYCKRDYIRLYGIKCAKCN

Branchiostoma_floridae IHDQYILRVAPDLEWHAACLKCSDCNQYLDETCTCFVREGKTYCKRCYVRLFGTKCAKCS

Ciona_intestinalis IHDQYILRVAPNLEWHAGCLKCADCGQYLDETCTCFVRDGKTYCKRDYTRLFGTKCNKCG

Drosophila_melanogaster IHDQYILRVAPDLEWHAACLKCQECRQFLDESCTCFVRDGKTYCKRDYVRLFGTKCDKCG

Homo_sapiens IGFSKNDFVMRARSKVYHIECFRCVACSRQLIPGDEFALREDG-LFCRADHDVVERASLG

Mus_musculus IGFSKNDFVMRARSKVYHIECFRCVACSRQLIPGDEFALREDG-LFCRADHDVVERASLG

Danio_rerio IGFSKNDFVMRARSKVYHIECFRCVACSRQLIPGDEFALREDG-LFCRADHDVVERATMG

Gallus_gallus IGFSKNDFVMRARAKVYHIECFRCVACSRQLIPGDEFALREDG-LFCRADHDVVERASLG

Xenopus_laevis IGFSKNDFVMRARSKVYHIECFRCVACSRQLIPGDEFALREDG-LFCRADHDVVERASLG

Branchiostoma_floridae LGFTKNDFVMRAKNKIYHIDCFRCVACSRQLIPGDEFALREDG-LFCKADHEVLERASNN

Ciona_intestinalis LCFSKNDFVMRARDKIYHIQCFKCVACSRQLIPGDEFALRDDG-LFCKADHEVATSGDMM

Drosophila_melanogaster NSFSKNDFVMRAKTKIFHIECFRCSACARQLLPGDEFALRDAGALYCKEDHDVLEKSSQS

Homo_sapiens AGDPL-SPL---HPAR------------PLQMAAEPIS-ARQPA----LRPHVHKQ-PEK

Mus_musculus AGDPL-SPL---HPAR------------PLQMAAEPIS-ARQPA----LRPHVHKQ-PEK

Danio_rerio AGDPL-SPL---HPAR------------PLQMAAEPIS-ARQPA----LRPHVHKQ-PEK

Gallus_gallus AGDPL-SPL---HPAR------------PLQMAAEPIS-ARQPA----LRPHVHKQ-PEK

Xenopus_laevis GSDPL-SPL---HPGR------------PLQMAAEPIC-ARQPA----LRPHVHKQ-PEK

Branchiostoma_floridae VDSNG-RAS---LGST------------DLEMATRPESHGRSDQ----RRPQVHKQDGHK

Ciona_intestinalis VHDGHMIPG---IPQTPNPQ-----GVISPQMGGERVISHRSGG----HSGGQRRSKDAK

Drosophila_melanogaster SLTSSSVESNNNISSSNNNNTNLSNNNHSSELGSMSDSGSESGSHKSIRDKRPSGPSDGK

Homo_sapiens TTRVRTVLNEKQLHTLRTCYAANPRPDALMKEQLVEMTGLSPRVIRVWFQNKRCKDKKRS

Mus_musculus TTRVRTVLNEKQLHTLRTCYAANPRPDALMKEQLVEMTGLSPRVIRVWFQNKRCKDKKRS

Danio_rerio TTRVRTVLNEKQLHTLRTCYNANPRPDALMKEQLVEMTGLSPRVIRVWFQNKRCKDKKRS

Gallus_gallus TTRVRTVLNEKQLHTLRTCYAANPRPDALMKEQLVEMTGLSPRVIRVWFQNKRCKDKKRS

Xenopus_laevis TTRVRTVLNEKQLHTLRTCYAANPRPDALMKEQLVEMTGLSPRVIRVWFQNKRCKDKKRS

Branchiostoma_floridae PTRVRTVLNEKQLHTLRTCYAANPRPDALMKEQLVEMTGLSPRVIRVWFQNKRCKDKKKS

Ciona_intestinalis TTRVRTVLNEKQLHTLRTCYAANCRPDALMKEQLTEMTGLSSRVIRVWFQNKRCKDKKRS

Drosophila_melanogaster PTRVRTVLNEKQLHTLRTCYNANPRPDALMKEQLVEMTSLSPRVIRVWFQNKRCKDKKKT

Homo_sapiens IMMKQLQQQQ----------------------PNDKTNIQGMTGTPMVAASPERHDGGLQ

Mus_musculus IMMKQLQQQQ----------------------PNDKTNIQGMTGTPMVAASPERHDGGLQ

Danio_rerio ILMKQLQQQQ----------------------PNDKTNIQGMTGTPMVATSPERHDGGLQ

Gallus_gallus IMMKQLQQQQ----------------------PNDKTNIQGMTGTPMVAASPERHDGGLQ

Xenopus_laevis ILIKQLQQQQ----------------------PNDKTNIQGMTGTPMVASSPERHDGGLQ

Branchiostoma_floridae ILMKQMQEQASKQDLSSDSIVWDTLPDDMTASPNGMSGIGRLNGVPMVAQEPVRHESQMQ

Ciona_intestinalis IALKQIQEQQAKQQHNNE-------------QGNNVQGLSGMNGVPMVASEPVRNDNSVS

Drosophila_melanogaster IQMKLQMQQE---------------------KEGRKLGYGAMQGIPMIASSPVRHDSPLN

Homo_sapiens ANPVEVQSYQ--PPWKVLSDFALQSDID--------QPAFQQL-----VNFSEGGPGSNS

Mus_musculus ANPVEVQSYQ--PPWKVLSDFALQSDID--------QPAFQQL-----VNFSEGGPGSNS

Danio_rerio ANQVEVQSYQ--PPWKVLSDFALQSDID--------QPAFQQL-----VNFSEGGPGSNS

Gallus_gallus ANPVEVQSYQ--PPWKVLSDFALQSDID--------QPAFQQL-----VNFSEGGPGSNS

Xenopus_laevis ANPVEVQTYQ--PPWKVLSDFALQSDID--------QPAFQQLTCFSQVNFSEGGPGSNS

Branchiostoma_floridae ANPVEVQSYQQPPPWKALSDFALQSDIE--------QPAFQQLKSGDVHAQLPCGGGMMV

Ciona_intestinalis VAPVEVRNYQQ-PAWKALSDFALQSEIE--------QPAFQQLMNN----FSDQGQGSIS

Drosophila_melanogaster LQGLDVQTYQ--PPWKALSDFALHADLDSNGAINTHTPAFQQLVNQMHG-YDL--NGMPI

Homo_sapiens TG------SEV--ASMSSQLPDTPNSM----VA---SPIEA-------------------

Mus_musculus TG------SEV--ASMSSQLPDTPNSM----VA---SPIEA-------------------

Danio_rerio TG------SEV--ASMSSQLPDTPNSM----VA---SPIEA-------------------

Gallus_gallus TG------SEV--ASMSSQLPDTPNSM----VA---SPIEA-------------------

Xenopus_laevis TG------SEV--ASMSSQLPDTPNSM----VA---SPIEA-------------------

Branchiostoma_floridae LTLHPHSHTPV--SSFDHNSGNVPTSQPGPGMAAPGGPGEANGNGPGVPPPGGPEMPVGA

Ciona_intestinalis DSSEISSIPSVSSASMDSTTCSTPHTVESS------VPACS-------------------

Drosophila_melanogaster LPPHPHSHPAQGP----------PHQH----PPPPGGPHNHQNQQPNQ-QPGGSSLDSGI

Homo_sapiens ------------------------------------------------------------

Mus_musculus ------------------------------------------------------------

Danio_rerio ------------------------------------------------------------

Gallus_gallus ------------------------------------------------------------

Xenopus_laevis ------------------------------------------------------------

Branchiostoma_floridae SPPNPVTCT---------------------------------------------------

Ciona_intestinalis ------------------------------------------------------------

Drosophila_melanogaster TSHHHPDSTDSYVTYLESDDKSKLALTPSSSSSASAGTSISSPPSGVGAGGGGAVGGGSG

Homo_sapiens -----------------------------------

Mus_musculus -----------------------------------

Danio_rerio -----------------------------------

Gallus_gallus -----------------------------------

Xenopus_laevis -----------------------------------

Branchiostoma_floridae -----------------------------------

Ciona_intestinalis -----------------------------------

Drosophila_melanogaster VLGLGVVANQSATEQLMQMLQKVTGSASPASHAVL
